# Hypoxia modulation by dual-drug nanoparticles for enhanced synergistic sonodynamic and starvation therapy

**DOI:** 10.1186/s12951-021-00837-0

**Published:** 2021-03-26

**Authors:** Jingxue Wang, Ju Huang, Weichen Zhou, Jiawen Zhao, Qi Peng, Liang Zhang, Zhigang Wang, Pan Li, Rui Li

**Affiliations:** 1 Department of Ultrasound, The Third Affiliated Hospital, Chongqing Medical University, Chongqing, 400010 People’s Republic of China; 2 Chongqing Key Laboratory of Ultrasound Molecular Imaging, Institute of Ultrasound Imaging, The Second Affiliated Hospital, Chongqing Medical University, Chongqing, 400010 People’s Republic of China; 3 University-Town Hospital, Chongqing Medical University, Chongqing, 401331 People’s Republic of China

**Keywords:** Hypoxia, Sonodynamic therapy, Starvation therapy, Dual-modal imaging, Nanomedicine

## Abstract

**Background:**

Sonodynamic therapy (SDT) is an emerging non-invasive therapeutic technique. SDT-based cancer therapy strategies are presently underway, and it may be perceived as a promising approach to improve the efficiency of anti-cancer treatment. In this work, multifunctional theranostic nanoparticles (NPs) were synthesized for synergistic starvation therapy and SDT by loading glucose oxidase (GOx, termed G) and 5,10,15,20-tetrakis (4-chlorophenyl) porphyrin) Cl (T (p-Cl) PPMnCl, termed PMnC) in Poly (lactic-co-glycolic) acid (PLGA) NPs (designated as MG@P NPs).

**Results:**

On account of the peroxidase-like activity of PMnC, MG@P NPs can catalyze hydrogen peroxide (H_2_O_2_) in tumor regions to produce oxygen (O_2_), thus enhancing synergistic therapeutic effects by accelerating the decomposition of glucose and promoting the production of cytotoxic singlet oxygen (^1^O_2_) induced by ultrasound (US) irradiation. Furthermore, the NPs can also serve as excellent photoacoustic (PA)/magnetic resonance (MR) imaging contrast agents, effectuating imaging-guided cancer treatment.

**Conclusion:**

Multifunctional MG@P NPs can effectuate the synergistic amplification effect of cancer starvation therapy and SDT by hypoxia modulation, and act as contrast agents to enhance MR/PA dual-modal imaging. Consequently, MG@P NPs might be a promising nano-platform for highly efficient cancer theranostics.

**Graphical Abstract:**

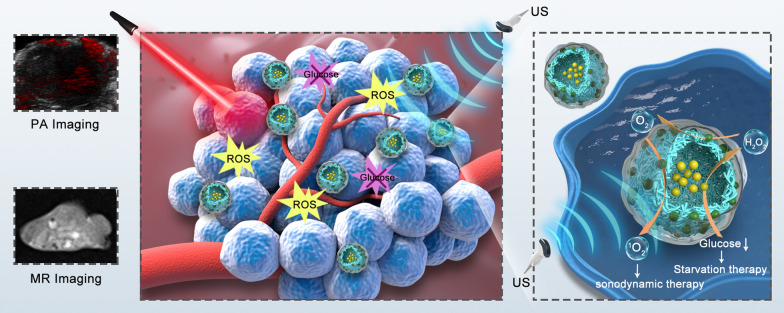

**Supplementary Information:**

The online version contains supplementary material available at 10.1186/s12951-021-00837-0.

## Background

Cancers pose a severe threat to human health [1]. Despite the beneficial effects of reported cancer therapeutic modalities (e.g., surgery, chemotherapy and radiotherapy), their severe side effects, such as the high risk of tissue damages, immune-system impairment and therapeutic inefficiency, usually cause failure of cancer therapy [2–4]. Therefore, it is in pressing need to explore efficient and non-invasive therapeutic modalities for cancer treatments.

In recent years, modulating cancer metabolism emerges as a representatively appealing therapeutic modality for cancer therapy [5–8]. Most tumor cells require sufficient nutrients and energy to support their rapid tumor metabolism and proliferation. Even in the presence of normal O_2_ levels, cancer cells generally produce energy through anaerobic glycolysis (the Warburg effect), which enhances the cellular sensitivity to changes in intracellular glucose concentration [7, 9]. Given this feature of cancer cells, GOx, which can effectively oxidize glucose into gluconic acid and hydrogen peroxide (H_2_O_2_), has gained attention in cancer starvation therapy [10–13]. However, it is challenging to block off glucoses present in the tumor regions due to the constant supply from tumor blood vessels. In order to improve treatment efficacy, GOx-guided starvation therapy should be in concert with other therapeutic modalities.

SDT is a promising approach to eliminate solid tumors, as it efficiently induces the apoptosis and death of the cancer cells by activating localized sonosensitizer molecules to generate reactive oxygen species (ROS) [14–17]. Compared to photodynamic therapy (PDT) [18, 19], SDT provides more advantages including deeper tissue penetration, less side effects and lower cost [17, 20–22]. SDT is fueled by oxygen, and consequently, hypoxia in tumor microenvironment would mitigate the efficacy of oxygen-dependent sonodynamic therapy. There is a consensus in recent studies that sonosensitizer plays a crucial role in affecting the efficacy of SDT. The tremendous development of porphyrins and their derivatives in SDT has been witnessed attributed to their unique properties, such as their large π-electron conjugated system, catalytic performance and broad-ranging optoelectronic [23–26]. It is notable that metalloporphyrins have distinct bioinorganic functions due to their particular chemical properties. For instance, Mn- and Fe-porphyrins have been recognized as potential superoxide dismutase mimic for anticancer therapy [27–29]; ^64^Cu-porphysomes have been validated as an efficient positron emission tomography (PET) agent[30, 31]; Mn (II)—and Mn (III)—porphyrin were used in T1-weighted MR contrast imaging [32]. However, the poor water solubility, fast metabolism, and potential photosensitive toxicity of metalloporphyrins limited their clinical application.

In general, imaging-guided cancer therapy has attracted broad attention [33, 34]. High spatial resolutions and deep tissue penetration are the primary features of some diagnostic-image technologies, such as photoacoustic (PA) imaging, computed tomography (CT) imaging and magnetic resonance (MR) imaging [35–37]. The optimum therapeutic time can be determined by monitoring the distribution of drugs using these imaging techniques. Thus, therapeutic contrast agents essentially enable imaging-guided cancer treatment.

**Scheme 1:**
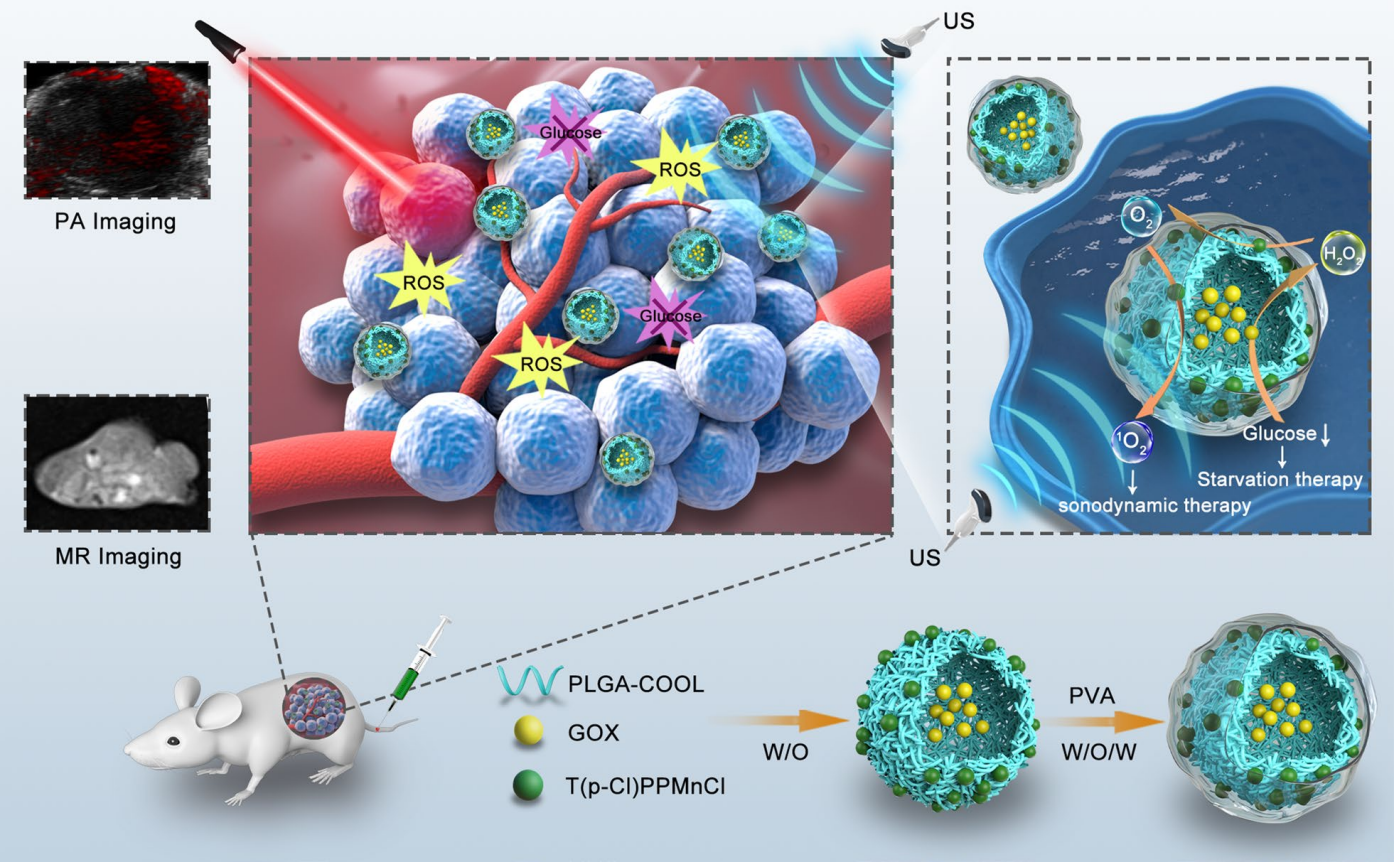
The preparation route of MG@P NPs and Schematic illustration of the multifunctional NPs for MR/PA dual imaging-guiaded synergistic amplification therapy

Herein, multifunctional theranostic nanoparticles (NPs) were synthesized for dual-modal imaging (PA/MR imaging) guided synergistic starvation therapy and SDT. Poly (lactic-co-glycolic) acid (PLGA), a well-known biodegradable and biocompatible polymer approved by the United States Food and Drug Administration [38, 39], served as the carrier to load glucose oxidase (GOx, termed G) and Mn (5,10,15,20-tetrakis (4-chlorophenyl) porphyrin) Cl (Mn, termed PMnC). GOx, an endogenous oxido-reductase, is widely distributed in living organisms and characterized by its inherent biocompatibility and non-toxicity [6, 40]. As GOx efficiently catalyzes glucose oxidation and produce gluconic acid and H_2_O_2_ in tumor region in presence of O_2_, cancer cells fail to access to sufficient O_2_ and nutrients to sustain their fast growth, resulting in cell death. PMnC, a hydrophobic metal-porphyrin complex, was used as sonosensitizer, thus the nano-complex could genarate abundant ROS upon US irradiation. Meanwhile, PMnC can be used for PA/MR dual-modal imaging on account of its unique chemical properties. Inspired by the peroxidase-like activity of the Mn-HPyP modified liposomes [41], PMnC was also demonstrated to catalyze the H_2_O_2_ to produce O_2_, which accelerates the decomposition of glucose. Furthermore, the generated O_2_ could improve efficacy of oxygen-dependent SDT. Therefore, the multifunctional MG@P NPs can effectuate the synergistic amplification effect of image-guided cancer starvation therapy and SDT by hypoxia modulation, and effectively inhibit tumor growth (Scheme 1).

## Materials and methods

### Materials and reagents

The poly (lactic-co-glycolic) acid (PLGA) (50:50, MW: 12,000) was obtained from Daigang BIO Engineer Co., Ltd. (Shan Dong, China). Mn (5,10,15,20-tetrakis (4-chlorophenyl) porphyrin) Cl (T (p-Cl) PPMnCl, denoted as PMnC) was purchased from Shanghai Macklin Biochemical Co., Ltd. (Shanghai, China). Glucose Oxidase (GOx) and poly (vinyl alcohol) (MW: 25,000) were obtained from Sigma-Aldrich (St. Louis, MO, USA). One Step TUNEL Apoptosis Assay Kit, 1,1-dioctadecyl-3,3,3′,3′-tetramethylindocarbocyanine (DiI), 4,6-Diamidino-2-phenylindole (DAPI) and 2,7-dichlorodihydrofluoresceindiacetate (DCFH-DA) were purchased from Beyotime Biotechnology (China). Bradford Protein Assay Kit was obtained from Beijing Solarbio Science & Technology Co., Ltd. (China). Singlet Oxygen Sensor Green (SOSG) was purchased from Thermo Fisher Scientific (USA). CCK-8, Calcein-AM and propidium iodide (PI) were obtained from Dojindo (Japan). Roswell Park Memorial Institute-1640 complete medium (RPMI-1640) was purchased from Shanghai Zhongqiaoxinzhou Biotech. (Shanghai, China). Indocyanine green (ICG) was obtained from Aikeda Chemical Reagent Co., Ltd (Chengdu, China).

## Methods

### Synthesis of MG@P NPs

PLGA encapsulating PMnC and GOx (termed MG@P NPs) were performed via a previously reported double emulsion [15]. Firstly, 2 mg PMnC were added into 50 mg PLGA that pre-dissolved in 3 mL dichloromethane (CH_2_Cl_2_). Next, 10 mg GOx dissolved in 200 μL double distilled water was added to above mixture as the stock solution. Subsequently, the mixture was emulsified using a probe sonicator (Sonics & Materials, Inc., USA) at an intensity of 65 W for 3 min. For the second emulsion, 8 mL 4% poly (vinyl alcohol) water solution were added into the above emulsified solution and homogenized using the same sonicator at an intensity of 40 W for 2 min. Then, 10 mL of a 2% isopropyl alcohol water solution were added to the as-prepared emulsion. And the emulsion was then stirred by a magnetic stirrer at 100 rpm for 3 h to remove CH_2_Cl_2_ in a well-ventilated fumefood. Finally, the pellets of MG@P NPs were collected after a centrifugation process (rcf = 10,000 × g, 5 min). The preparation of M@P NPs was fabricated similar to the above process, except that aqueous solution (200 μL) containing GOx was replaced by double distilled water (200 μL). The same method also applied to the synthesis of GOx-PLGA NPs (G@P NPs) and ICG-PLGA NPs (I@P NPs) and the addition amount of GOx and ICG was 10 and 2 mg respectively.

### Characterization of MG@P NPs

Transmission electron microscope (TEM, Hitachi-7500, Japan) and Scanning electron microscope (SEM, Hitachi S-3400 N, Japan) were applied to detect the morphology and structure of the MG@P NPs. Area-elemental mapping was adopted to confirm the existence of Mn in NPs on FEI-Talos F200S electron microscope. The confocal laser scanning microscope (CLSM, A1R; Nikon, Tokyo, Japan) was used to observe the DiI-stained MG@P NPs. The particle diameters and zeta potential were determined using a Malvern Zetasizer instrument (Nano ZS90, UK). The colloidal stability of MG@P NPs dissolved in phosphate-buffered solution (PBS) were monitored at 1, 2, 3, 4, 5, 6 and 7 days, respectively. UV–vis spectrophotometer (UV-2600, Shimadzu, Japan) was used to determine the UV–vis absorption spectra of PMnC, GOx and MG@P NPs. The amount of encapsulated PMnC, GOx and ICG were monitored using the UV–vis spectrometer, Bradford Protein Assay Kit and multimode reader (SpectraMax M3), respectively. The encapsulation efficiency (EE) was then calculated using a standard curve by plotting the absorbance against concentration. A fluorescence spectrophotometer (RF-5301PC, Shimadzu, Japan) was used to quantitatively evaluate ^1^O_2_ generation of MG@P NPs in vitro. A certain amount of SOSG dissolved in methanol was added to MG@P NPs suspensions (10 μg/mL). The mixture was activated by ultrasound (US) for different durations (0, 30, 60, 90, 120, 150 and 180 s, respectively). Under the US irradiation, SOSG emit fluorescence at 525 nm after capturing ^1^O_2_. And the fluorescence intensity was recorded by fluorescence spectrophotometer.

### Cell culture and animals

The murine mammary carcinoma (4T1) cells were obtained from the Chinese Academy of Sciences. The cells were incubated in RPMI-1640 complete medium and cultured at 37 °C in a humidified atmosphere containing 5% CO_2_. Healthy female BALB/c mice (6–8 weeks old) were obtained from the Experimental Animal Center of Chongqing Medical University. All the animal experiments were approved by the Animal Ethics Committe of Chongqing Medical University. For the establishment of 4T1 tumor xenograft, subcutaneous tissues in the right flank of each mouse were injected with the 1 × 10^6^ 4T1 cells suspended in 100 μL PBS solution.

### Catalytic activity measurements

MG@P NPs-induced pH change was measured in the presence of glucose at a concentration of 1 mg/mL. Briefly, glucose (1 mg/ mL) was mixed with GOx, MG@P NPs (GOx = 10 μg/mL) or M@P NPs (the concentration is the same as that of MG@P NPs). The real-time pH values of the solutions were measured with a pH meter (PHB-4, Shanghai INESA Scientific Instrument Co., Ltd, China). 3,3′,5,5′-tetramethyl-benzidine (TMB), horseradish peroxidase (HRP) and glucose were used to analyze the catalytic activity of GOx in NPs. Multimode reader (SpectraMax M3) was applied to monitor the chromogenic reaction at 640 nm. Due to the interference of PMnC at 640 nm, G@P NPs was used to measure the enzyme activity. The reaction process is as follows:

$${\text{Glucose}}\;\xrightarrow{{{\text{GOx}}}}\;{\text{Gluconic}}\;{\text{acid}} + {{\text{H}}_2}{{\text{O}}_2}$$

$${{\text{H}}_2}{{\text{O}}_2}\xrightarrow{{{\text{HRP}}}} \cdot {\text{OH}}$$

$${\text{colorless TMB}} + \cdot {\text{OH}} \to {\text{chromogenic TMB cation (blue)}}$$

To investigate the oxygen generation ability through H_2_O_2_ catalyzed by PMnC and M@P NPs, the dissolved oxygen concentration in M@P NPs, PMnC solutions (PMnC = 38.4 μg/mL), H_2_O@P NPs (only water-loaded PLGA) and PBS was measured in the presence of H_2_O_2_ (100 mM) using a dissolved oxygen meter in real-time. To mimic the decomposition of hydrogen peroxide by M@P NPs in the tumor microenvironment, 4T1 cells were seeded in petri dishes. After 20 h, the culture medium was replaced with H_2_O@P NPs and M@P NPs solutions, which were diluted to equal concentrations (100 μg/mL) in RPMI-1640 containing H_2_O_2_ (1 mM). The addition of RPMI-1640 was referred to as the control group. Then, oxygen partial pressure electrode (JPBJ-608, Shanghai INESA Scientific Instrument Co., Ltd, China) was placed into the solutions to record the change of dissolved oxygen concentration in the medium and the dish was immediately sealed with liquid paraffin to avoid oxygen exchange. The rate reduction of dissolved oxygen (DO%) was calculated as follows: ${\text{DO\% = }}\frac{{{\text{D}}{{\text{O}}_{0}}-{\text{ D}}{{\text{O}}_{\text{t}}}}}{{{\text{D}}{{\text{O}}_{0}}}}{\text{*100\% ,}}$ where DO_0_ represented the initial values and DO_t_ represented the measured values at time t. In addition, 4T1 tumor-bearing BALB/c mice were used to evaluate the tumor hypoxia. After intravenously injecting 300 μL M@P NPs or saline for 24 h, tumors were excised for determining the expression of hypoxia inducible factor-1α (HIF-1α) by immunofluorescence staining. PA images were collected to measure oxygen saturation (sO_2_) in tumor region pre-injection and 24 h after injection.

### Cellular uptake behaviors of MG@P NPs

For intracellular uptake observation, 4T1 cells (5 × 10^4^/dish) were seeded in confocal laser scanning microscopy (CLSM) dishes for 24 h and co-incubated with DiI-labeled MG@P NPs (10 μg/mL, 1 mL) for different time points (3, 6 and 12 h). Then, the confocal dishes were washed with PBS three times, and the cells were fixed with 4% paraformaldehyde (1 mL) for 15 min, and dyed with DAPI (200 μL) for 15 min. Finally, cellular uptake were observed using CLSM. To qualitatively evaluate the efficiency of intracellular uptake, 4T1 cells were cultured in 12-well plates at a density of 5 × 10^4^ cells per well and co-incubated with DiI-MG@P NPs for different durations (3, 6 and 12 h). Then, the treated 4T1 cells were collected for analysis of cellular uptake behaviors by flow cytometry (BD FACSVantage SE, USA).

### Intracellular ROS generation of MG@P NPs

4T1 cells seeded in CLSM dishes at a density of 5 × 10^4^ cells per dish were divided into five groups: control group (Control), US only group (US), G@P NPs group (G@P NPs), M@P NPs combined with US irradiation group (M@P NPs + US) and MG@P NPs combined with US irradiation (MG@P NPs + US). After 24 h incubation, the cells were co-incubated with different NPs (G@P, M@P and MG@P NPs) dispersed in RPMI-1640 at the same PLGA concentration of 20 μg/mL. After 12 h of coincubation with the corresponding NPs, the dishes were rinsed with PBS and DCFH-DA was added. After 20 min, cells in the US, M@P + US and MG@P + US groups were exposed to US irradiation (1 MHz, 1.5 W/cm^2^) for 60 s. The fluorescence of the above samples was visualized by CLSM. The fluorescence intensities in the abovementioned groups were further determined by flow cytometry.

### In vitro synergistic therapeutic performance

4T1 cells were seeded in 96-well plates at a density of 1 × 10^4^ cells per well for 20 h. These cells were randomly divided into the following groups: control group (Control), US only group (US), G@P NPs group (G@P NPs), M@P NPs combined with US irradiation group (M@P NPs + US) and MG@P NPs combined with US irradiation (MG@P NPs + US). Then, different NPs (G@P, M@P, and MG@P NPs) dispersed in RPMI-1640 were added to the wells at PLGA concentrations of 20 and 40 μg/mL. After 12 h coincubation, cells in US, M@P + US and MG@P + US groups were exposed to US irradiation (1 MHz, 1.5 W/cm^2^) for 60 s. After half an hour, a standard CCK-8 assay was used to evaluate the cell viabilities via a plate reader.

To further examine the effect of synergistic therapy, 4T1 cells were seeded in 12-well plates at a density of 5 × 10^4^ cells per well for 20 h. The cells were divided into five groups: control group (Control), US only group (US), G@P NPs group (G@P NPs), M@P NPs combined with US irradiation group (M@P NPs + US) and MG@P NPs combined with US irradiation (MG@P NPs + US). Then, the cells were incubated with different NPs (G@P, M@P, and MG@P NPs) dispersed in RPMI-1640 at PLGA concentrations of 20 μg/mL. After 12 h, cells in US, M@P + US and MG@P + US groups were exposed to US irradiation (1 MHz, 1.5 W/cm^2^) for 60 s. Finally, cell apoptosis level of the aforementioned groups was evaluated using flow cytometry. Furthermore, to visually observe the therapeutic effects after various treatments by CLSM, CAM and PI dye were managed to dye living (green fluorescence) and dead (red fluorescence) cells, respectively.

### In vitro and in vivo MR and PA Imaging of MG@P NPs

For in vitro PA imaging, a serial concentration of 0.5, 1.0, 2.0, 4.0 and 8.0 mg/mL were used for PA signal detection and to evaluate the linearity of the PA signal as a function of MG@P NPs and I@P NPs concentration. The PA images were obtained by the VEVO LASR PA imaging system (VIVO 2100; FUJIFILM Visual Sonics, Inc., Canada). For in vitro MR imaging, the prepared MG@P NPs solution at the concentrations of 0.5, 1.0, 2.0, 4.0 and 8.0 mg/mL were placed in plastic tubes and serial T1-weighted MR scans were collected using fast low-angle shot two-dimensional gradient-echo sequence (Siemens Magnetom Skyra 3.0 T, Germany). The signal intensities were measured for each sample in the region of interest (ROI).

To evaluate the accumulation of MG@P NPs in tumors, the T1-weighted MR imaging and PA imaging (λ_ex_ = 690 nm) of 4T1 tumor-bearing mice were performed. After intravenously injecting 300 μL (10 mg/mL) MG@P NPs emulsions at different time points (2, 4, 8, 24 and 48 h), the corresponding PA and MR images were acquired.

### Evaluation of MG@P NPs accumulation in tumor

4T1 tumor-bearing mice (n = 15) were intravenously injected with MG@P NPs (300 μL, 10 mg/mL) in saline. Mice were then sacrificed at varied time points (2, 4, 8, 24 and 48 h). The PMnC content in the tumors was quantitatively determined by inductively coupled plasma mass spectrometry.

### In vivo antitumor capacity

When the volume of the tumor reached 80 mm^3^, twenty-five BALB/c mice were randomly assigned into five groups (Control, US, G@P, M@P + US, MG@P + US) to evaluate the in vivo therapeutic performance. After intravenous injection of 300 μL (10 mg/mL) different NPs for 24 h, the regions of the tumors were irradiated by US (1 MHz, 3 W/cm^2^) for 5 min. The mice in the US only group were only exposed to US (3 W/cm^2^, 5 min) without the NPs injection. The treatments were carried out every 4 days over 16 days. On day 16, the mice were sacrificed, and the tumors were collected. Tumor volumes and body weights of the mice were recorded. Tumors were then fixed with 4% polyoxymethylene for histological analysis including H&E, TUNEL and PCNA (H&E: hematoxylin–eosin staining; TUNEL: TdT-mediated dUTP nick-end labeling; PCNA: proliferating cell nuclear antigen). And major organs were fixed with 4% polyoxymethylene for H&E staining. According to the weight of the tumors, the tumor inhibition rate was determined.

### Biosafety assay

For blood glucose assays, female BALB/c mice (n = 5) were intravenously injected with 300 μL of MG@P NPs. At 0.25, 0.5, 1, 2, 3, 4, 5, 6, and 8 h, blood was collected by nicking the tail vein gently and tested by a glucometer (Accu-chek performa, Roche, Switzerland).

Twenty-five healthy BALB/c mice were intravenously injected with MG@P NPs (300 μL, 10 mg/mL). The control group (n = 5) was administered with saline only. The mice were euthanized after a certain time (1, 3, 7, 14, and 28 day post-injection), and blood of mice was collected for blood biochemistry and blood routine examinations. The major organs of mice were excised and then fixed with 4% polyoxymethylene for H&E staining.

### Statistical analysis

Statistical analysis was performed with SPSS 26.0 software. Data were presented as the mean ± standard deviation. The significance of the data was analyzed according to the Student’s t-test: *P < 0.05.

## Results and discussion

### Synthesis and characterization of MG@P NPs

**Fig. 1:**
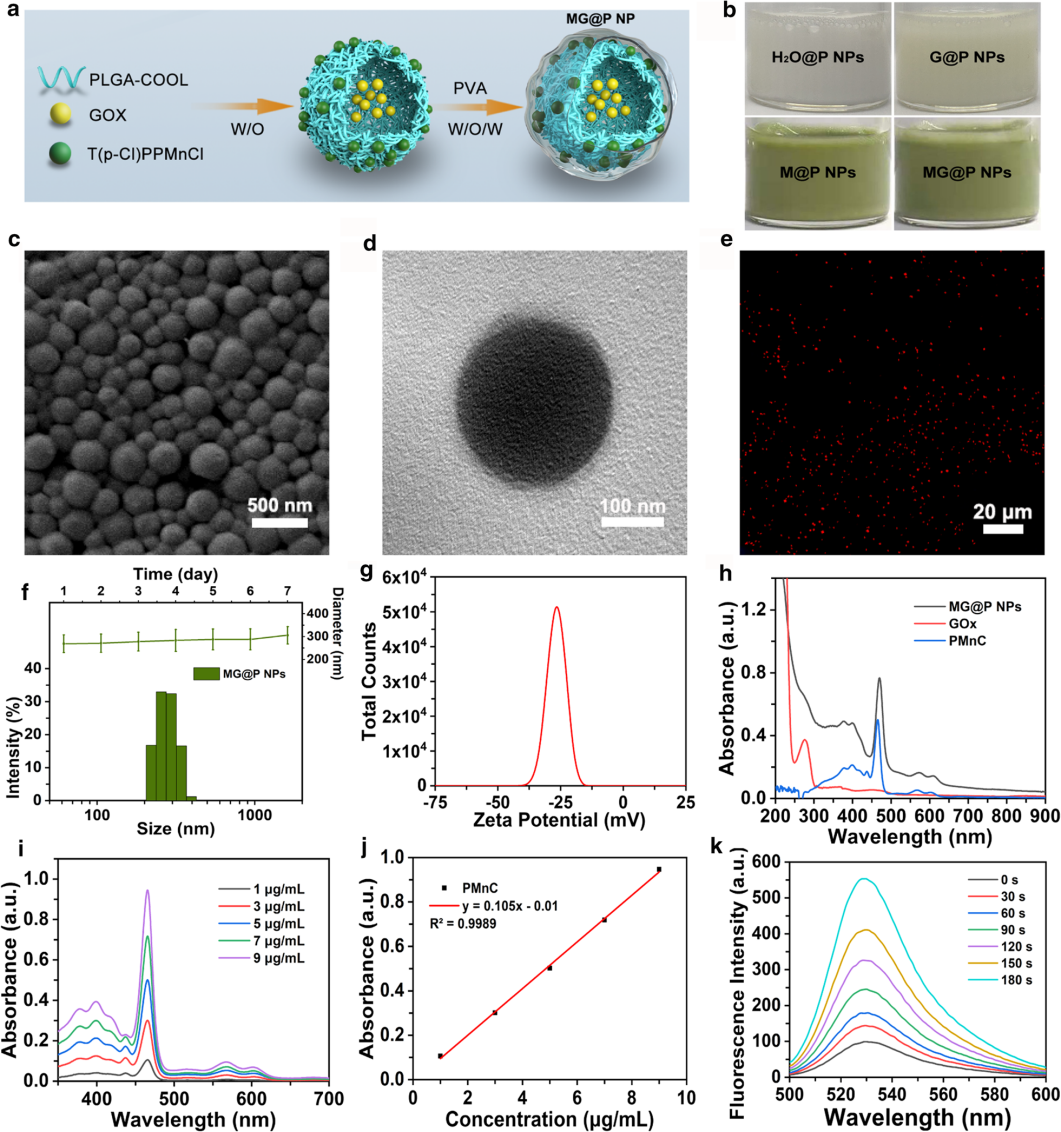
Characterizations of MG@P NPs. **a** Schematic diagram for the fabrication of MG@P NPs. **b** Photographs of H_2_O@P NPs, G@P NPs, M@P NPs and MG@P NPs dispersed in PBS. **c** SEM and **d** TEM of MG@P NPs. **e** CLSM image of DiI-stained MG@P NPs (red fluorescence). **f** Size distribution of MG@P NPs and the change of average size with prolonged time duration. **g** Zeta potential of MG@P NPs. **h** UV − vis absorbance spectra of free PMnC, free GOx, and MG@P NPs. **f** UV–vis spectra of PMnC in different concentrations and **j** linear relationship between concentration of MG@P NPs and absorbance. **k** Time-dependent SOSG absorption spectra of MG@P NPs (10 µg/mL) under US irradiation

PLGA was used to carry one hydrophobic material (PMnC) in the shell and one hydrophilic material (GOx) in the core to form shell-core structured NPs (designated MG@P NPs) via double emulsification method (Fig. 1a). The appearance of the NPs solutions changed from milky white to dark green when PMnC was loaded, and the color changed from milky white to light yellow when GOx was loaded (Fig. 1b), which demonstrated the successful loading of PMnC and GOx. First, the obtained MG@P NPs displayed well-defined spherical shape and homogenous in size, as revealed by SEM (Fig. 1c) and TEM images (Fig. 1d). The high-angle annular dark-field (HAADF) STEM image (Additional file 1: Figure S1) of MG@P NPs also showed spherical shape. And the area-elemental mapping analysis of the NPs revealed the existence of Mn element, illustrating the successful loading of PMnC in MG@P NPs (Additional file 1: Figure S1). In addition, the MG@P NPs which labeled with DiI in PLGA shell exhibited strong red florescence, as detected by CLSM (Fig. 1e).

The mean diameters of the MG@P NPs were 278.3 ± 40.96 nm (Fig. 1f). To check the colloidal stability of the nanoparticles under certain electrolyte solutions, the changes in size of MG@P NPs in PBS within 7 days were measured. Figure 1f showed that there were no significant changes in size within seven days. In addition, the zeta potential of MG@P NPs was found to be − 26.7 ± 3.29 mV (Fig. 1g). The negative zeta potential may facilitate the nanodroplets to repel each other and prevent aggregation in vivo [42]. Besides, by decreasing the clearance of the reticuloendothelial system (RES) the negative zeta potential nanoparticles with negative surface charges could prolong circulation time of MG@P NPs in the blood, which is beneficial to the delivery of these NPs [6].

The UV–vis absorption spectra of PMnC showed almost the identical curves as those of the MG@P NPs (Fig. 1h), indicating a large amount of PMnC in the resultant after loading PMnC. The UV−vis absorption intensity of PMnC showed a concentration-dependent manner (Fig. 1i). According to the standard curve and relative absorbance intensity of PMnC in the UV−vis spectrum (Fig. 1j), the encapsulation efficiency of PMnC in MG@P NPs was calculated to be 95.83 ± 1.35%. The encapsulation efficiencies of GOx measured by Bradford Protein Assay Kit was 23.80 ± 1.96%. The encapsulation efficiency of ICG in I@P NPs measured by multimode reader (SpectraMax M3) was 66.45 ± 2.81%. To investigate the SDT efficiency, SOSG assay was carried out. Under US irradiation, these NPs generated ^1^O_2_ and the intensities increased with the extension of irradiation time (Fig. 1k). The results indicated that these MG@P NPs could act as potential sonosensitizers for SDT.

### In vitro catalytic activity measurements

**Fig. 2:**
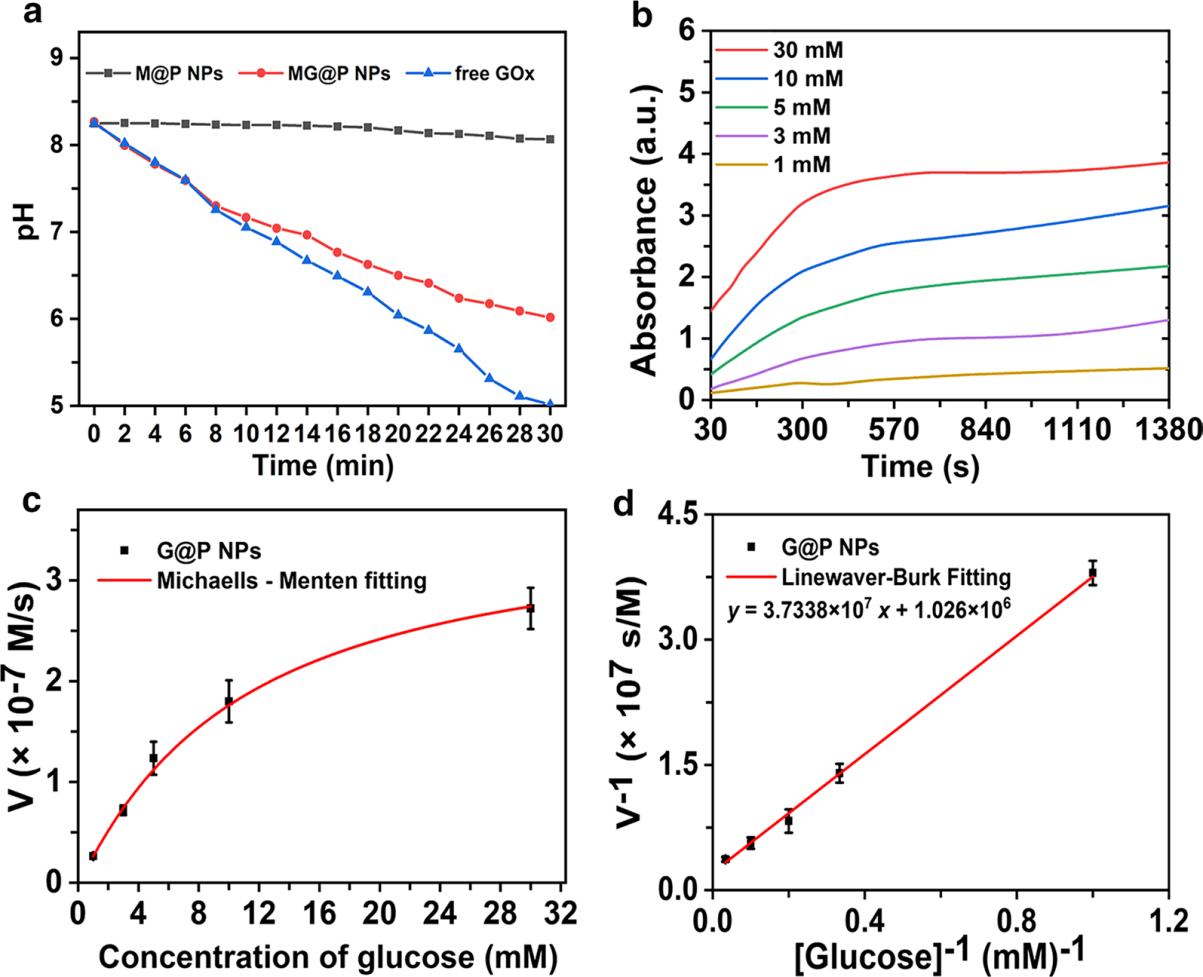
Catalytic activity measurements of GOx. **a** The pH value changes of glucose solution with the addition of M@P NPs, MG@P NPs or free GOx. **b** Time-course absorbance of G@P NPs upon the addition of different concentrations of β-D-glucose (1, 3, 5, 10 and 50 mM). Michaelis Menten kinetics (**c**) and Lineweaver Burk fitting of G@P NPs

The catalytic decomposition of glucose by GOx would reduce the pH of the reaction system owing to the production of gluconic acid [43, 44]. As illustrated in Fig. 2a, the pH values of the glucose solution remained constant at approximately 8.19 with M@P NPs treatment. In contrast, due to glucose decomposition by GOx, a dramatic pH drop (from 8.28 to 5.07 and 8.28 to 6.12) happened in free GOx and MG@P NPs groups, respectively. The result strongly indicated the catalytic ability of GOx after encapsulation was retained. However, the pH reduction in the free GOx group was more obvious than the MG@P NPs group, since organic solvents (CH_2_Cl_2_) and emulsification methods (ultrasound) during the preparation of nanoparticles may reduce the enzyme activity. Then, TMB assay was applied to further prove the functionalities of GOx [45, 46]. To exclude the effect of PMnC on absorbance at 640 nm, G@P NPs were used to verify the catalytic properties of GOx encapsulated in PLGA. As shown in Fig. 2b, at a certain concentration of NPs, the absorbance of the mixture with various concentrations of glucose (1–30 mM) revealed an increase as the concentration of glucose increased. All initial velocities (v_0_) were calculated according to the average initial velocities of absorbance changes, and then Michaelis–Menten curves and Lineweaver–Burk plot were obtained (Fig. 2c, d). The Michaelis–Menten constant (K_M_) and maximum catalytic velocity (V_max_) values were calculated to be 10.53 mM and 3.62 × 10^–7^ M/s for G@P NPs. The K_M_ for GOx in PLGA NPs demonstrated that GOx attained 50% of the maximum catalytic efficiency in the presence of 10.53 mM glucose. The results showed that the GOx in PLGA NPs performed a desirable therapeutic effect, since the concentration of glucose in most cancer cells is far below 20 mM [47]. The corresponding equations were as follows:

$${\text{A = kbc}}$$

$${{\text{v}}_{0}}{\text{ = }}\frac{{{{\text{V}}_{{\text{max}}}}{\text{}} \cdot {\text{[S]}}}}{{{{\text{K}}_{\text{M}}}{\text{ + [S]}}}}$$

$$\frac{{1}}{{{{\text{v}}_{0}}}}{\text{ = }}\frac{{{{\text{K}}_{\text{M}}}}}{{{{\text{v}}_{{\text{max}}}}}} \cdot \frac{{1}}{{\left[ {\text{S}} \right]}}{\text{ + }}\frac{{1}}{{{{\text{v}}_{{\text{max}}}}}}$$.

**Fig. 3:**
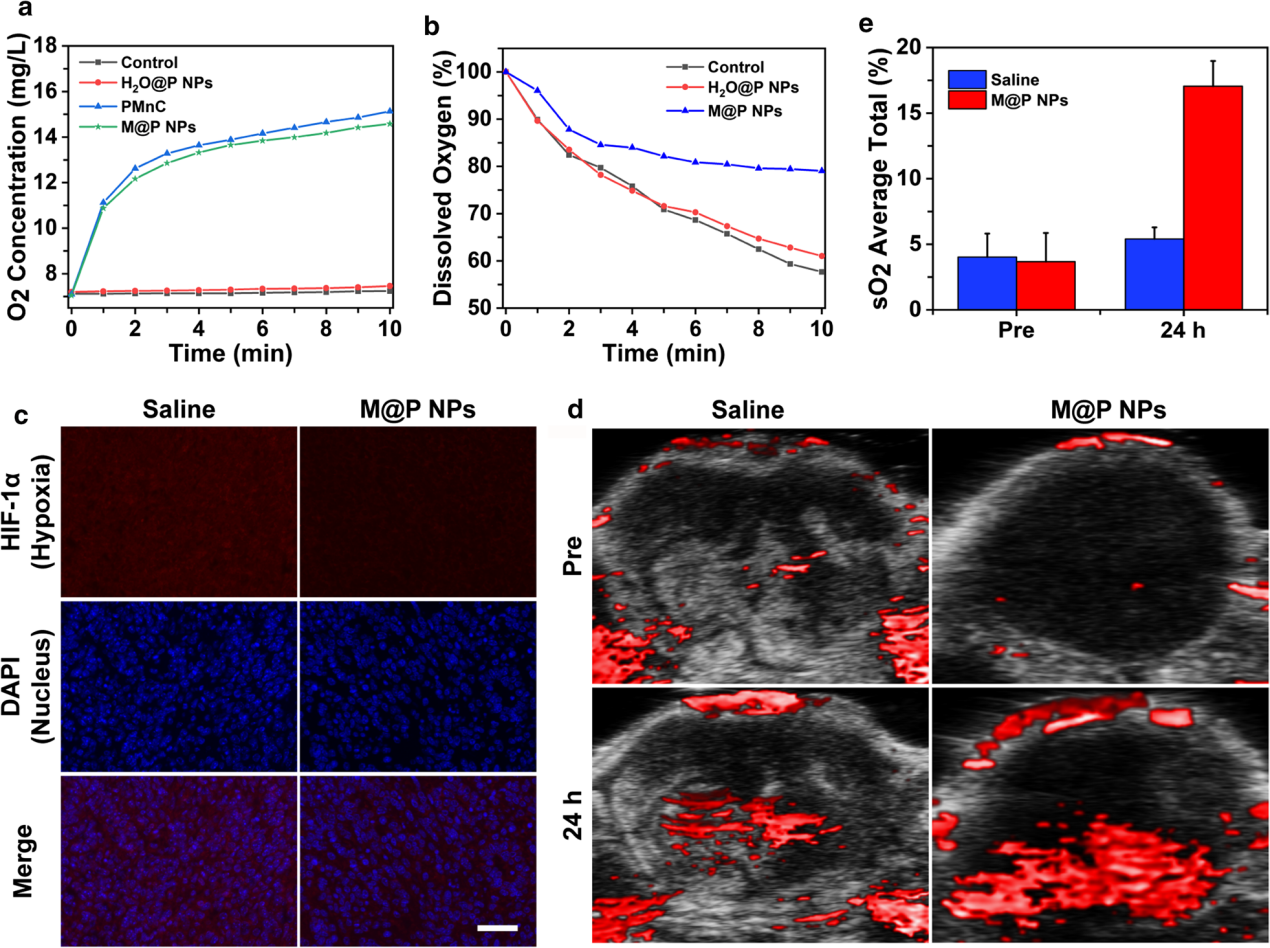
The capability of PMnC to modulate hypoxia. **a** The O_2_ concentration changes of H_2_O_2_ solution upon the addition of PBS, H_2_O@P NPs, free PMnC or M@P NPs. **b** Determination DO content of 4T1 cell after incubating with different NPs. The initial value was taken to be 100%. **c** Immunofluorescence staining images of tumor slices stained by the hypoxia probe at 24 h post injection of saline or M@P NPs. The scale bars are 50 μm. **d** PA images of 4T1 tumor-bearing mice with injection of saline or M@P NPs recorded in the Oxy-Hemo mode at pre and 24 h. **e** The semi-quantitative analysis of corresponding sO_2_ average total (%) values in tumor regions of figure (**d**)

To investigate the oxygen generation ability through M@P NPs-catalyzed H_2_O_2_ decomposition, a dissolved oxygen meter was used to monitor the oxygen concentration detection. Figure 3a illustrated the oxygen content changes among the 4 groups. Compared to the control and H_2_O@P NPs groups, the oxygen content of which was maintained at a low value, the M@P NPs and free PMnC groups saw an O_2_ increase from 7.05 to 14.58 and 7.1 to 15.13 mg/mL, respectively. As shown in Fig. 3b, the Control and H_2_O@P NPs groups exhibited the same decreasing tendency in DO%, and the DO% was significantly lower than that observed in the M@P NPs groups.

These results demonstrated that PMnC had the ability to catalyze H_2_O_2_, while PLGA as the carrier had no obvious contribution to the H_2_O_2_ decomposition. Therefore, M@P NPs had the potential to decompose H_2_O_2_ in the tumor microenvironment and elevate the relative oxygen concentration in tumor areas. HIF-1α produced by tumor cells plays a crucial role in an adaptive response to hypoxia by regulating gene expression, and it would be down-regulated when increasing tumor oxygenation [48, 49]. The red fluorescence of M@P NPs group was significantly lower than that of the saline group (Fig. 3c), indicating a decrease in the expression of HIF-1α.

sO_2_ is an essential indicator of tumor metabolism and therapeutic response [50], which can be measured by reconstructing the distribution of oxygenated and deoxygenated hemoglobin using the VEVO LASR PA imaging system. PA images of 4T1 tumor-bearing mice with the injection of M@P NPs recorded in the Oxy-Hemo mode showed a marked increase in the signal (Fig. 3d). Based on the semi-quantitative analysis of ROI in photoacoustic images, the sO_2_ average total (%) of the whole tumor site increased from 3.67% before M@P NPs injection to approximately 17.05%, while there was no significant change in the saline group (Fig. 3e), which further demonstrated the ability of M@P to reduce tumor hypoxia.

### Intracellular uptake

**Fig. 4:**
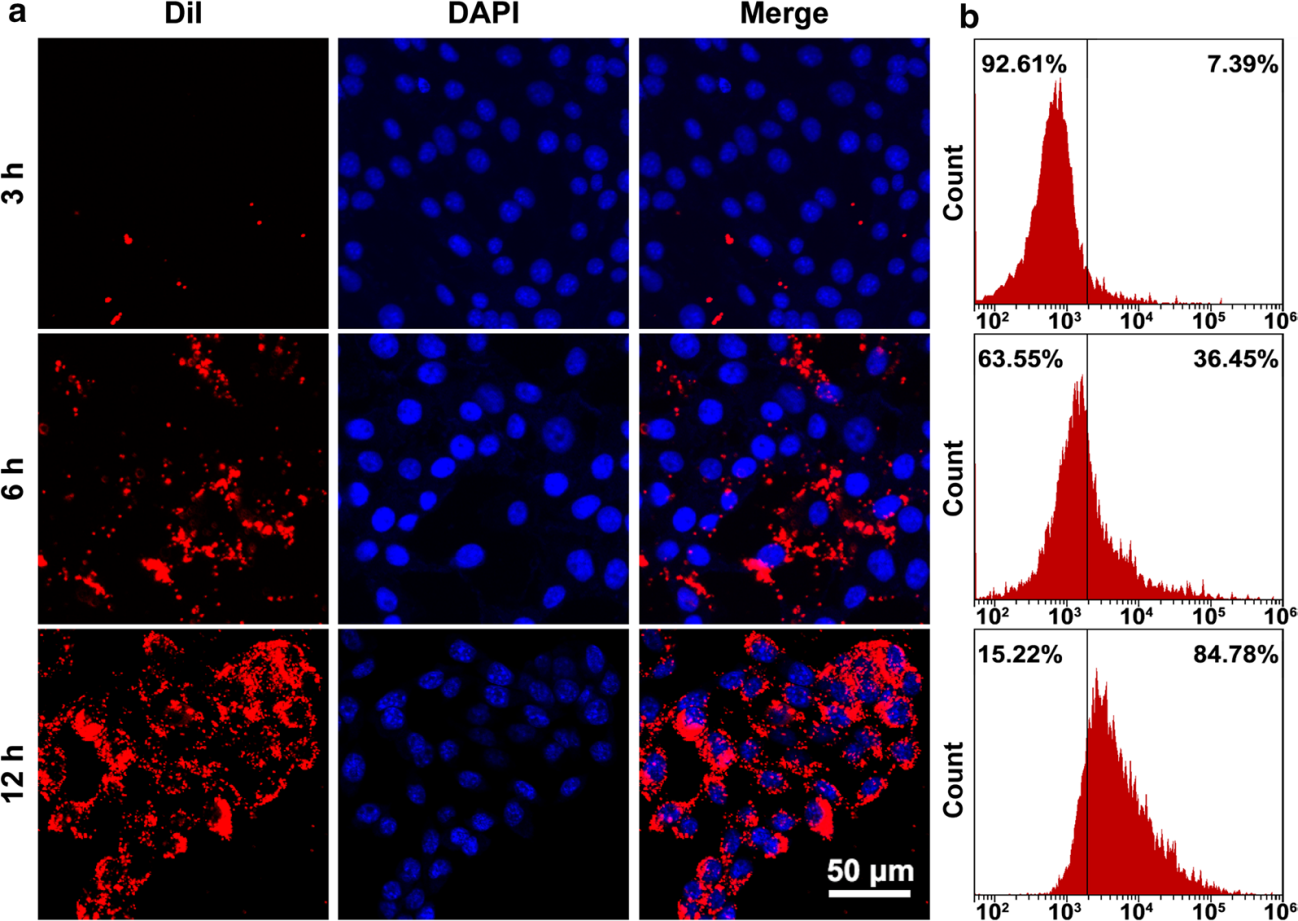
Cellular uptake behaviors of MG@P NPs. **a** CLSM images of 4T1 cells after incubation with DiI-stained MG@P NPs for elevated time (3 h, 6 h and 12 h). The scale bar is 50 μm. **b** Flow cytometry analysis of intracellular uptake of DiI-stained MG@P NPs

CLSM images showed that the DiI-MG@P NPs presented red fluorescence. The efficiency of phagocytosis was measured by the red fluorescence of the NPs. The fluorescence signal visible in 4T1 cells increased with the extension of incubation time (Fig. 4a). Flow cytometry was further used to quantitatively analyze the intracellular uptake of MG@P NPs. The results detected by flow cytometry were in accordance with CLSM observations (Fig. 4b), further demonstrating the affinity of MG@P NPs to tumor cells.

### In vitro ROS production of MG@P NPs

**Fig. 5:**
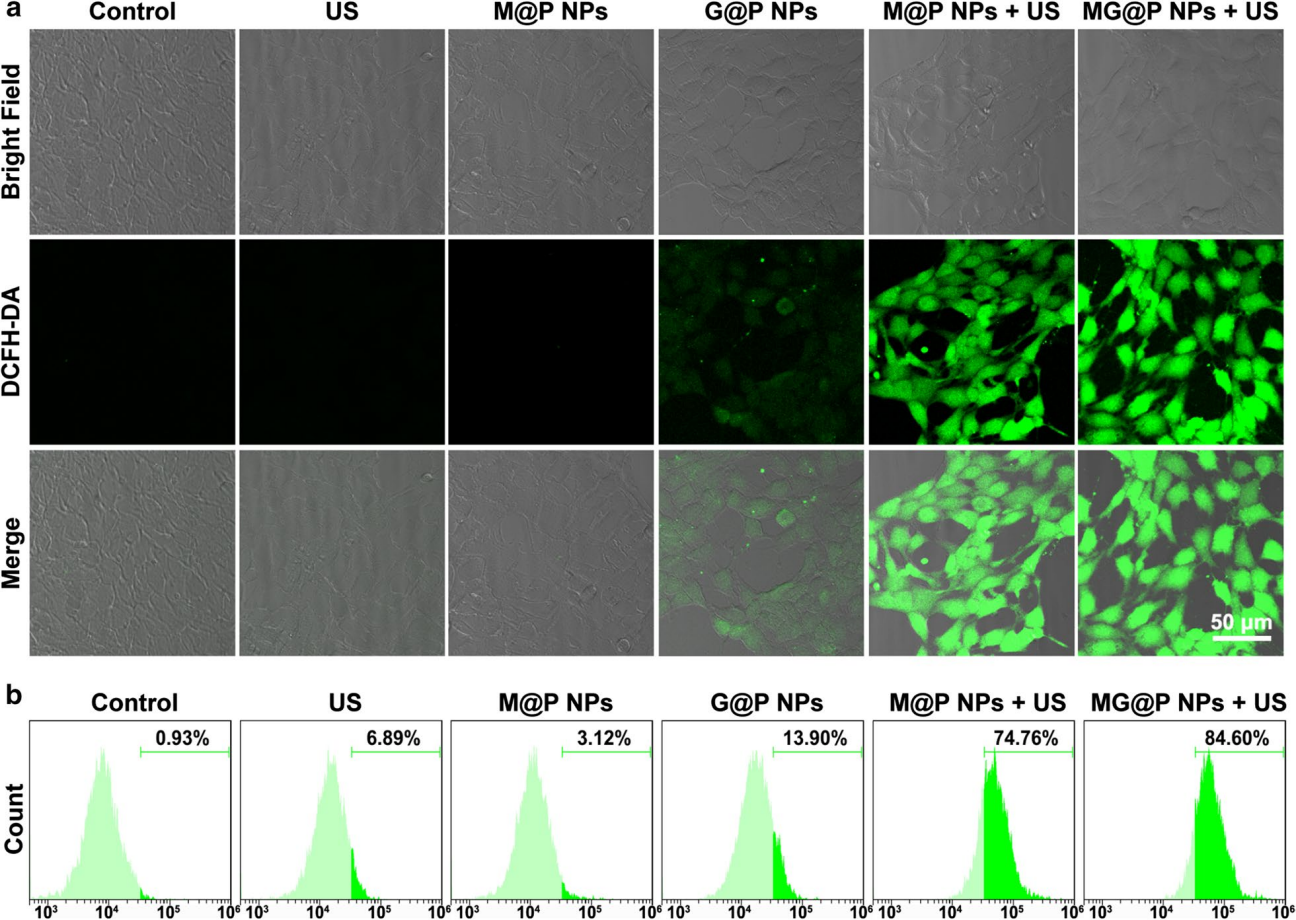
Intracellular ROS generation of MG@P NPs. **a** Intracellular ROS generation capacity illustrated by CLSM images of 4T1 cells. The scale bar is 50 μm. **b** The fluorescence intensity of 4T1 cells after incubation with DCFH-DA for different treatments detect by flow cytometry

The level of ROS production is essential for effective killing of tumor cells by SDT [51]. Given that PMnC is one of the promising forms of sonosensitizers, DCFH-DA, as a non-fluorescent precursor of DCF, was applied to detect the production of ROS by PMnC in this study. According to Fig. 5a, abundant ROS was generated in M@P NPs + US and MG@P NPs + US groups. Relatively weak fluorescence appeared in the G@P NPs group, which might be related to H_2_O_2_ produced by the GOx-mediated oxidation of glucose [40, 52]. There was only little green fluorescence in other groups. Flow cytometry assay was carried out to analyze the generation of ROS production quantitatively. The strongest fluorescence intensity was observed in the MG@P NPs + US group (81.65%), which was slightly higher than M@P NPs + US group (74.76%). Meanwhile, no obvious fluorescence generation was detected in other groups, indicating that the ROS was primarily generated only when PMnC was activated by US (Fig. 5b). These findings showed that PMnC could generate substantial ROS under US radiation in tumor cells, suggesting the potential of PMnC as a sonosensitizer.

### In vitro synergistic therapeutic efficacy

**Fig. 6:**
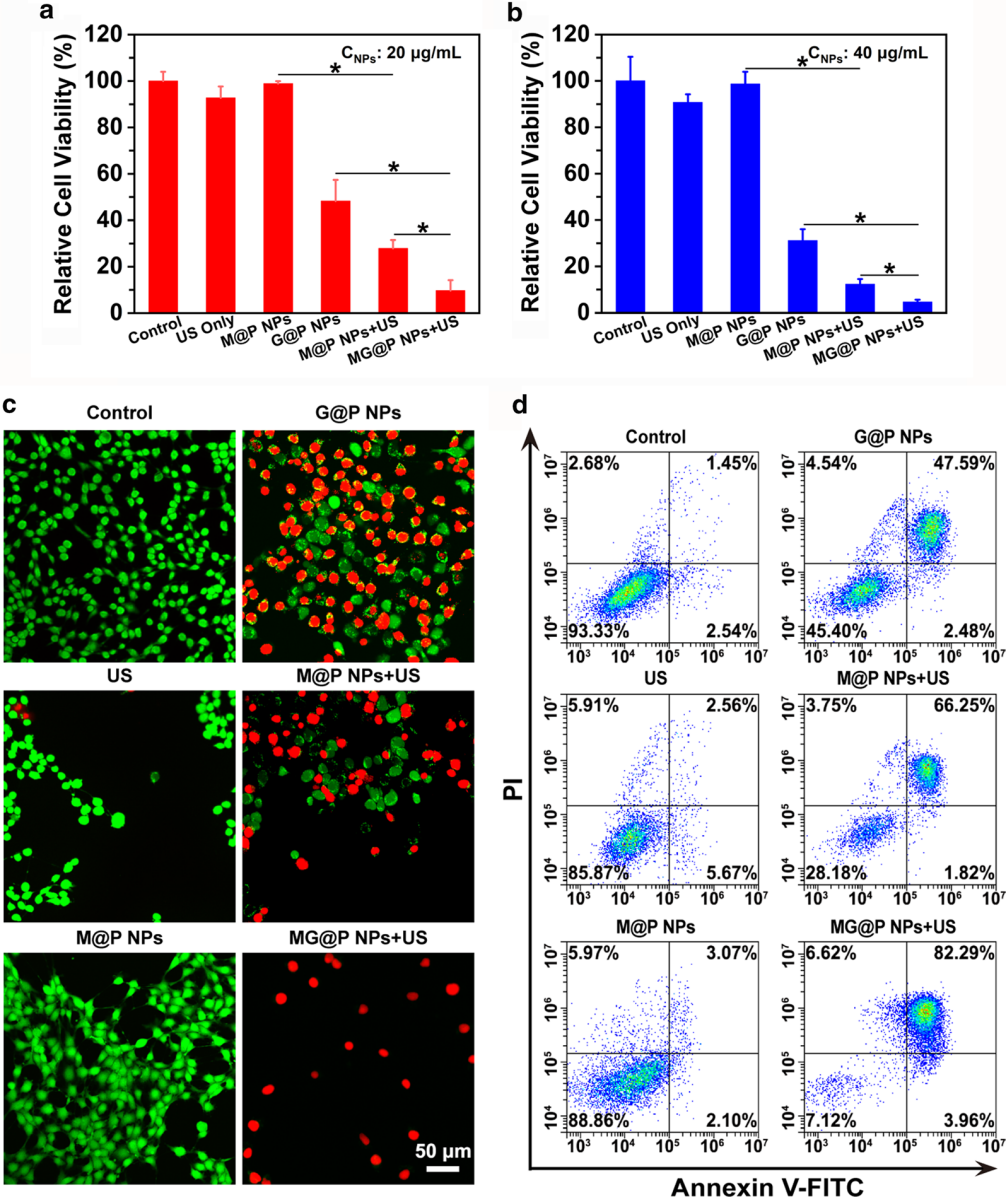
In vitro synergistic therapeutic performance. **a**, **b** The relative cell viability after varied treatments (concentration: 20 and 40 µg/mL) (n = 5) (*P < 0.05). **c** The therapeutic effects of various treatments detected by flow cytometry. The scale bar is 50 µm. **d** CLSM images of live/dead cells after different treatments

To verify the synergistic therapeutic efficacy of MG@P NPs in tumor cells, a CCK-8 assay was carried out. The cell viabilities showed concentration-dependent changes after various treatment (PLGA concentration: 20 and 40 μg/mL), suggesting that the high concentration of NPs exhibited greater cytotoxicity (Fig. 6a). At the identical concentration of NPs (20 μg/mL), 57.81 and 72.11% of cells in the G@P NPs group and M@P NPs + US group were damaged, respectively, while over 90% of cells treated with MG@P NPs + US group were damaged, showing the best tumor cell killing effect. Compare to the G@P NPs group and M@P NPs + US group, a significant lower cell viability was observed in MG@P NPs + US group, indicating that synergistic therapy had a better lethal effect than the mono-therapy (starvation therapy or SDT alone). The cell viability in the US group was consistently higher than 90%, demonstrating that Little cytotoxicity of US towards 4T1 cells. Subsequently, the fluorescence staining of living/dead cells showed the analogous cytotoxicity towards 4T1 cells, which further proved the excellent therapeutic efficacy of MG@P NPs (Fig. 6c). The quantitative analysis of apoptosis after various treatments were also obtained using a flow cytometry (Fig. 6d), and the results were consistent with results of the CCK-8 assay.

### MR/PA dual-modal imaging of the MG@P NPs in vitro and In vivo

**Fig. 7:**
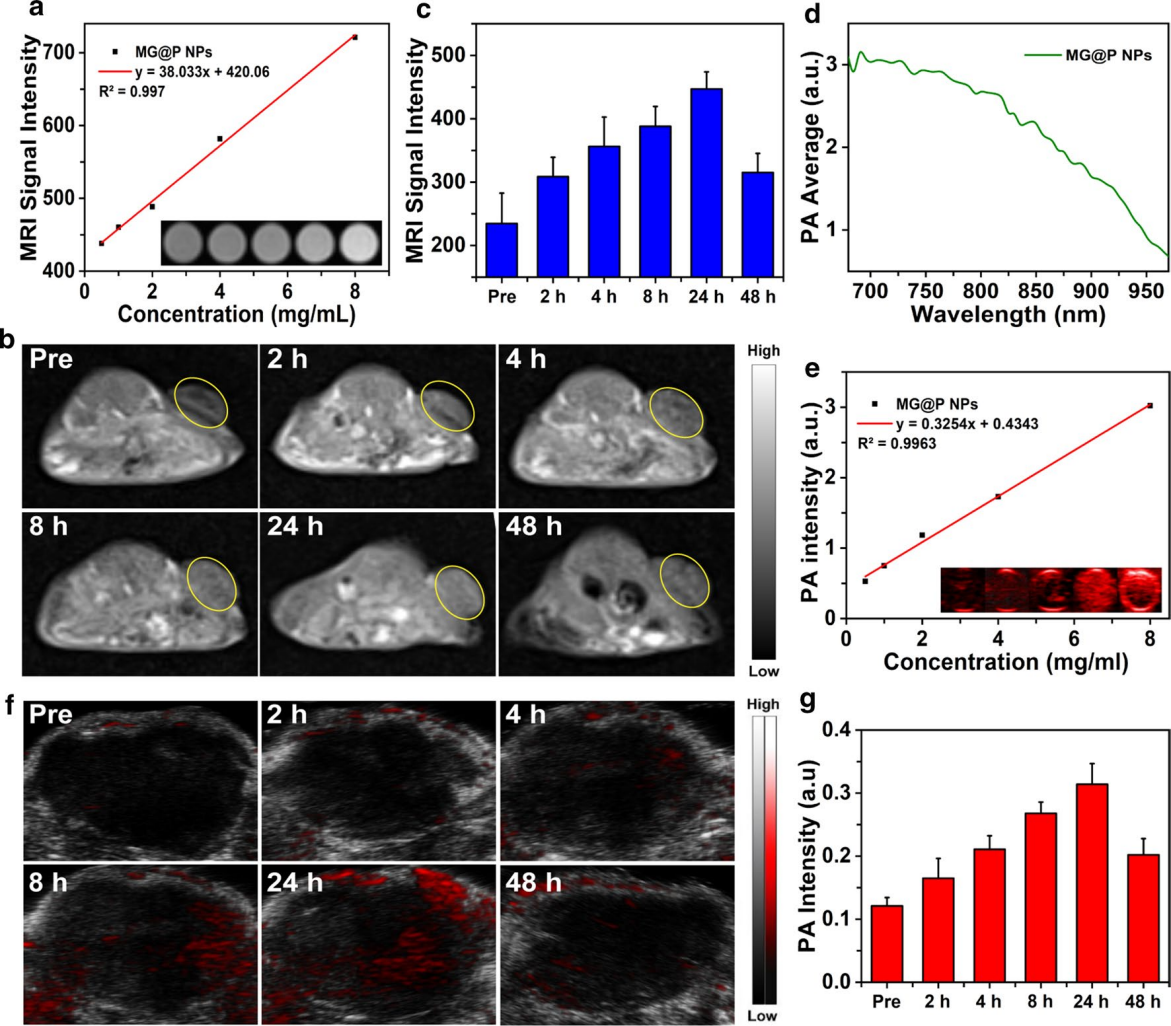
In vitro and in vivo MR/PA dual-modal Imaging **a** The linear relationship of T1 signal intensity and the concentration of MG@P NPs (0.5, 1.0, 2.0, 4.0 and 8.0 mg/mL); the inset was the T1-weighted MR images of MG@P NPs aqueous solutions at varied concentrations. **b** Time-dependent in vivo T1-weighted MR images of 4T1 tumor-bearing BALB/c mice after the intravenous injection of MG@P NPs. **c** Corresponding T1-weighted MR signal intensity of MG@P NPs in tumor varying with time. **d** Quantitative photoacoustic (PA) intensities of MG@P NPs in vitro. **e** The linear relationship of PA signal intensity and the concentration of MG@P NPs (0.5, 1.0, 2.0, 4.0 and 8.0 mg/mL); the inset was the PA images of MG@P NPs aqueous solutions at varied concentrations (λex = 690 nm). **f** Time-dependent PA images of 4T1 solid tumors after the intravenous injection of MG@P NPs. **g** The time-dependent PA signal intensity of MG@P NPs in tumor region

Potential diagnostic-imaging guidance is practically significant to monitor the accumulation of MG@P NPs in tumor regions. PMnC ion and its complexes have been developed as T1-MR imaging contrast agents[53]. Here, MG@P NPs showed an applicability for T1-MR imaging, and a good linear relationship was found between the MR signal intensity and the concentration of MG@P NPs (Fig. 7a). To evaluate the MR imaging enhancement capability in vivo, BALB/c mice bearing 4T1 tumors were intravenously injected with MG@P NPs. As time progressed, a T1 signal of tumor region showed an increasing trend within 24 h and then decreased (Fig. 7b). The semi-quantitated T1 signal intensity peaked at 24 h post-injection (Fig. 7c).

Furthermore, on account of the PA properties of porphyrins and their derivatives [20], MG@P NPs was also expected to be a PA contrast agent. MG@P NPs had the strongest PA signal at 690 nm with the excitation wavelength ranging from 680 to 970 nm (Fig. 7d). The in vitro PA images and signal intensities of MG@P NPs were measured. As shown in Fig. 7e, a good linear relationship was found between the PA signal intensities and the concentrations at the excitation wavelength of 690 nm. Indocyanine green (ICG), a typical PA agent [54], has been employed for comparison. ICG, a dye approved by the U.S. Food and Drug Administration (FDA), has been proven to convert absorbed optical energy into heat for PA imaging [55, 56]. A good linear relationship was found between the PA signal intensities and the concentrations at the excitation wavelength of 780 nm (Additional file 2: Figure S2). Due to the difference of drug loading, the concentration of PMnC was about 1.44 times as much as that of ICG at the same PLGA concentration. However, the PA signal intensity of I@P NPs was significantly higher than that of MG@P NPs at the same concentration of PLGA. The excitation wavelength limitation of PA imaging equipment weakened the PA imaging performance of MG@P NPs. But it also demonstrated the potential of MG@P NPs as a PA imaging contrast agent.

The in vivo PA imaging was performed on BALB/c mice bearing 4T1 tumors after intravenous administration of MG@P NPs. The highest PA signal of the tumor regions appeared at about 24 h, which was corresponded to the time of maximum signal appeared in MR imaging (Fig. 7f). Semi-quantitative PA signal intensity measurements concurrently showed an intensity change during the first 48 h (Fig. 7g). These results demonstrate the potential of MG@P NPs as a desirable MR/PA imaging contrast agent, which can achieve real-time imaging, diagnosis, and monitoring during tumor therapy.

### In vivo antitumor capacity

**Fig. 8:**
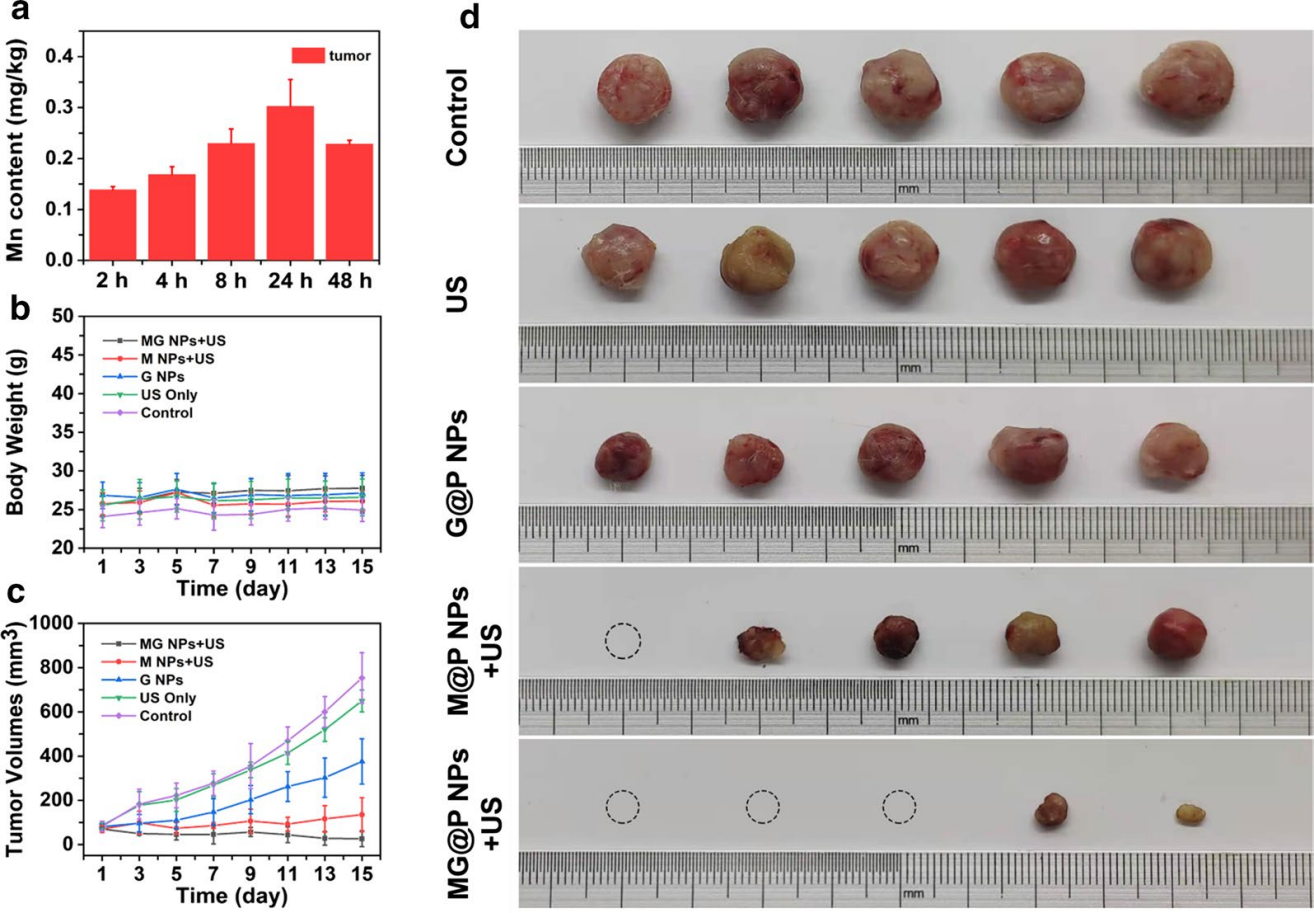
In vivo antitumor capacity. **a** Mn concentration in tumor site after the intravenous administration of MP@P NPs into tumor-bearing mice. Time-dependent body weights (**b**) and tumor volume curves (**c**) of 4T1-bearing mice of five groups after various treatments (n = 5). **d** Photographs of tumors dissected from mice in five groups after various treatments

To confirm the optimal timepoint of US irradiation following the injection, the accumulation of MG@P NPs in tumor region was analyzed by determining PMnC content over time after intravenous injection at 2, 4, 8, 24 and 48 h. The maximum content of PMnC in tumor regions was detected at 24 h (Fig. 8a). Next, twenty-five 4T1 tumor-bearing mice were randomly divided into five groups (Control, US, G@P NPs, M@P + US and MG@P + US) to test the in vivo synergistic therapeutic efficacy of MG@P NPs. Body weights and tumor volumes of the mice were monitored every two days to plot the growth curve (Fig. 8b, c). As shown in Fig. 8c, control and US groups increased rapidly during the entire treatment period, indicating that saline and US alone had no efficacy in restricting the growth of tumors. Nevertheless, different degrees of tumor growth suppression was observed in the other groups. The tumor growth of mice receiving starvation therapy (G@P) was increased approximately 4.62-fold compared with the original volumes due to the continuous supply of oxygen and nutrients in the capillaries. The tumor growth of mice in M@P + US group was remarkably suppressed, which is be related to the abundant generation of ROS during sonodynamic therapy. The synergistic treatment group (MG@P + US) performed best in inhibiting tumor growth compared with the single treatment groups. After 16 days of treatment, the tumors were excised for taking digital photographs (Fig. 8d).

**Fig. 9:**
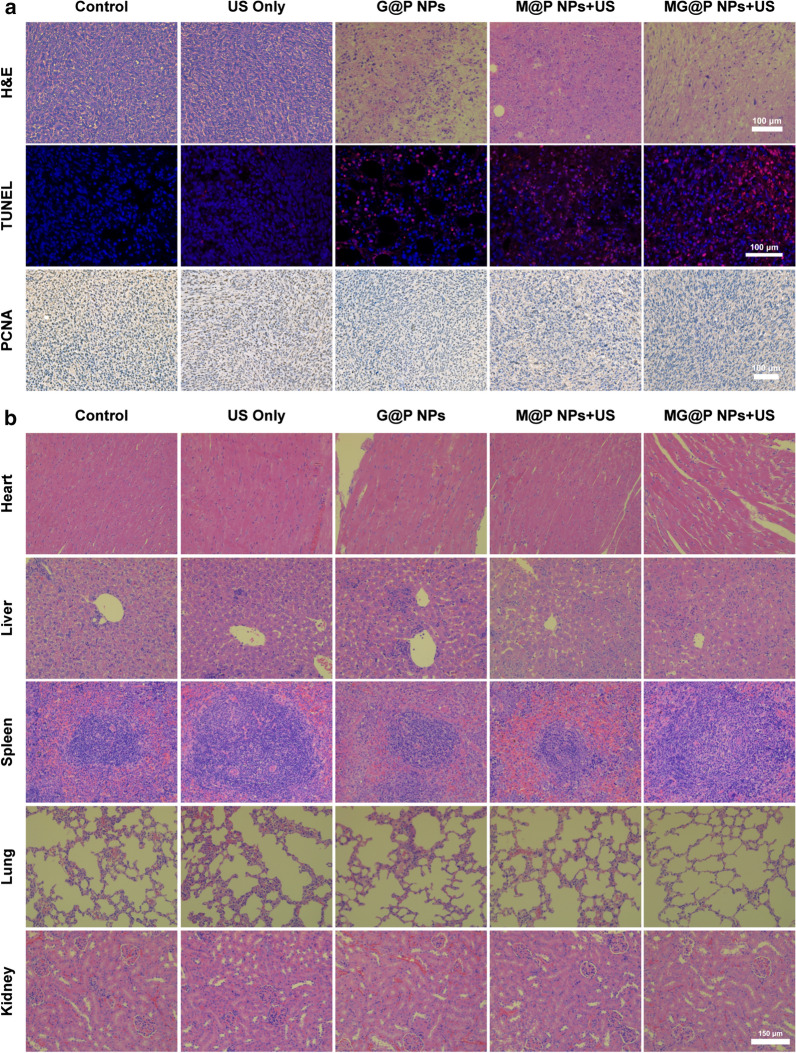
**a** H&E, TUNEL and PCNA staining of tumor sections after varied treatments. The scale bar is 100 μm. **b** H&E staining of major organs (heart, liver, spleen, lung, and kidney) after various treatments. The scale bar is 150 μm

To further evaluate necrosis and apoptosis of the tumor cells after various treatments, H&E, TUNEL, PCNA staining of tumors was performed (Fig. 9a). As shown in H&E staining, there was almost no necrosis in control and US group, while substantial damage and apoptosis of tumor cells in the group of MG@P NPs + US were found, which were much more remarkable compared to those in single treatment groups. Similarly, in TUNEL images, significant apoptosis (red fluorescence) was also observed in the MG@P + US group. The results of quantitative analysis of TUNEL staining were consistent with the above results (Additional file 3: Figure S3). The PCNA staining results evaluating proliferative activities of cancer cells (stained into brown) showed a lower proliferation index among G@P NPs, M@P NPs + US, and MG@P NPs + US groups, especially in MG@P NPs + US group. The results of H&E, TUNEL, and PCNA staining indicated satisfactory therapeutic outcomes of synergistic therapy. Furthermore, H&E staining of major organs of the mice, including the heart, liver, spleen, lung, and kidney, was conducted after the treatments to observe the corresponding pathological toxicity. It was clear that significant adverse effect on major organs was not found (Fig. 9b), suggesting the desirable therapeutic safety of the MG@P NPs in vivo.

### Biosafety assay of MG@P NPs

**Fig. 10:**
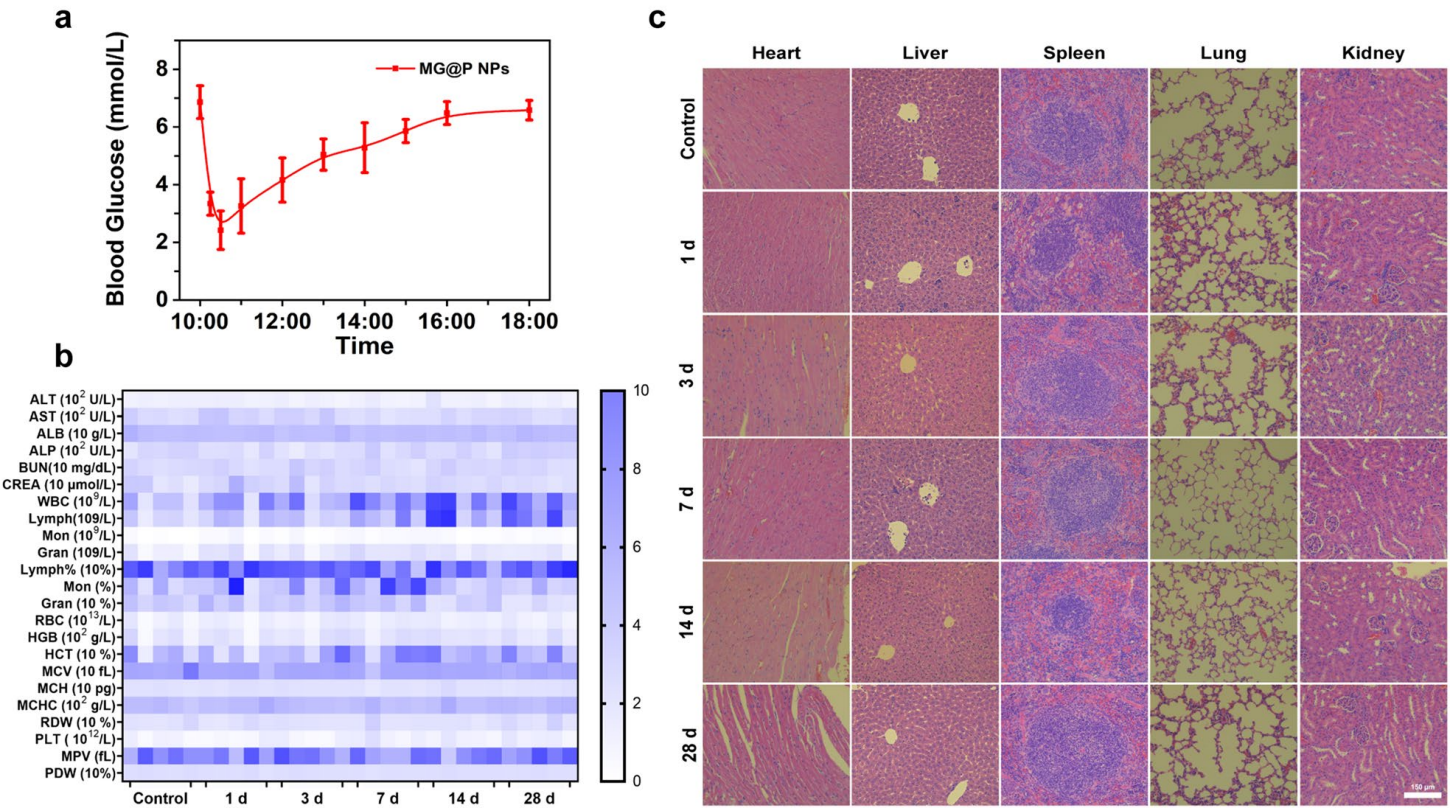
Biosafety assay of MG@P NPs. **a** The blood glucose of mice after intravenously injecting MG@P NPs (0, 0.25, 0.5, 1, 2, 3, 4, 5, 6 and 8 h). **b** Hematological and blood biochemical test of mice after intravenous injection of MG@P NPs (n = 5). **c** Images of H&E staining slices of major organs. The scale bar is 150 μm

The interferences of MG@P NPs in blood glucose were monitored with a glucometer. As shown in Fig. 10a, the blood glucose of mice after intravenous administration of MG@P NPs dropped to the lowest level at 0.5 h, and then gradually increased until it basically returned to normal level at 8 h, demonstrating that this starvation therapy had a slight reversible effect on blood glucose. In therapies mediated by nano-agents, most NPs enter tumor sites mainly by the enhanced permeability and retention (EPR) effect. Enhanced permeability of the tumor vasculature allows macromolecules to enter the tumor interstitial space, while the suppressed lymphatic filtration makes them stay there. The average size of MG@P NPs in this study endows these NPs with the capability of passive targeting to tumor tissues, which has been proved by PA/MR imaging and ICP detection. It has been proved that ultrasound can promote the drug release of PLGA nanoparticles [57]. Therefore, ultrasound irradiation on the tumor site can accelerate the release of GOx, and has little effect on the areas where there is no ultrasound irradiation.

Furthermore, the biosafety assessment by H&E staining of major organs was conducted. As shown in Fig. 10a, there were no significant adverse effects in various organs. Also, blood panel analysis and biochemistry assay were assayed at the end of experiments (Fig. 10b), and no detectable changes in blood indexes were texted. All results demonstrated the high safety of MG@P NPs in vivo.

## Conclusions

In summary, we successfully constructed a multifunctional theranostic nanoplatform (MG@P NPs) for MR/PA dual imaging-guided SDT and starvation therapy. Such an intriguing nanoplatform MR/PA dual-modal imaging contrast agent, achieving monitoring/guidance of the therapy delivery. The NPs had been demonstrated to generate abundant singlet oxygen under US irradiation and presented excellent SDT performance in vivo. Meanwhile, GOx locally consumed glucose to block the tumor nutrient supply, causing tumor cell starvation to further strengthen the treatment outcome. The generated O_2_ assisted by MG@P NPs further enhanced efficacy of oxygen-dependent SDT and starvation therapy. In addition, the desirable biosafety of these NPs guaranteed their further clinical translation. Therefore, this work reports a novel theranostic probe for tumor detection and treatment, which paves a promising way to achieve an ideal strategy for cancer therapy.

## Supplementary Information

**Additional file 1**: **Figure S1**. HAADF-STEM image and area-elemental mappings (C, O and Mn) of MG@P NPs.

**Additional file 2**: **Figure S2**. (a) Quantitative photoacoustic (PA) intensities of I@P NPs in vitro. (b) The linear relationship of PA signal intensity and the concentration of I@P NPs (0.5, 1.0, 2.0, 4.0 and 8.0 mg/mL); the inset was the PA images of I@P NPs aqueous solutions at varied concentrations (λex = 780 nm).

**Additional file 3**: **Figure S3**. The quantitative analysis of TUNEL staining.

## Data Availability

All data analyzed during this study are included in this published article and its supplementary information files.

## Abbreviations

SDT: Sonodynamic therapy
PLGA: Poly (lactic-co-glycolic) acid
GOx: Glucose oxidase
PMnC: Mn (5,10,15,20-tetrakis (4-chlorophenyl) porphyrin) Cl
PBS: Phosphate buffered saline
ICG: Indocyanine green
H_2_O_2_: Hydrogen peroxide
O_2_: Oxygen
^1^O_2_: Singlet oxygen
H_2_O: Double distilled water
CH_2_Cl_2_: Dichloromethane
DiI: 1,1-Dioctadecyl-3,3,3′,3′-tetramethylindocarbocyanine perchlorate
US: Ultrasound
PA: Photoacoustic
MR: Magnetic resonance
MG@P NPs: PMnC-GOx-PLGA NPs
M@P NPs: PMnC-PLGA NPs
G@P NPs: GOx-PLGA NPs
I@P NPs: ICG-PLGA NPs
DiI-MG@P NPs: DiI-PMnC-GOx-PLGA NPs
H_2_O@P NPs: H_2_O-PLGA NPs
SEM: Scanning electron microscope
TEM: Transmission electron microscope
HAADF: High-angle annular dark-field
CLSM: Confocal laser scanning microscope
FBS: Fetal bovine serum
CH_2_Cl_2_: Dichloromethane
SOSG: Singlet oxygen sensor green
TMB: 3,3′,5,5′-Tetramethyl-benzidine
DO: Dissolved oxygen
sO_2_: Oxygen saturation
DAPI: 4,6-Diamidino-2-phenylindole
ROS: Reactive oxygen species
DCFH-DA: 2,7-Dichlorodi-hydrofluoresceindiacetate
CCK-8: Cell Counting Kit-8
Calcein-AM: Calcein acetoxymethyl ester
PI: Propidium iodide
ROI: Region of interest
H&E: Hematoxylin–eosin staining
TUNEL: TdT-mediated dUTP nick-end labeling
PCNA: Proliferating cell nuclear antigen
