# Correction to: Hypoxia modulation by dual-drug nanoparticles for enhanced synergistic sonodynamic and starvation therapy

**DOI:** 10.1186/s12951-021-01038-5

**Published:** 2021-09-30

**Authors:** Jingxue Wang, Ju Huang, Weichen Zhou, Jiawen Zhao, Qi Peng, Liang Zhang, Zhigang Wang, Pan Li, Rui Li

**Affiliations:** 1 Department of Ultrasound, The Third Affiliated Hospital, Chongqing Medical University, Chongqing, 400010 People’s Republic of China; 2 Chongqing Key Laboratory of Ultrasound Molecular Imaging, Institute of Ultrasound Imaging, The Second Affiliated Hospital, Chongqing Medical University, Chongqing, 400010 People’s Republic of China; 3 University-Town Hospital, Chongqing Medical University, Chongqing, 401331 People’s Republic of China

## Correction to: J Nanobiotechnol (2021) 19:87 https://doi.org/10.1186/s12951-021-00837-0

Following publication of the original article [1], the authors identified an error in Fig. 10.

The original version of figure (Fig. 10) is provided in this correction.

**Fig. 10:**
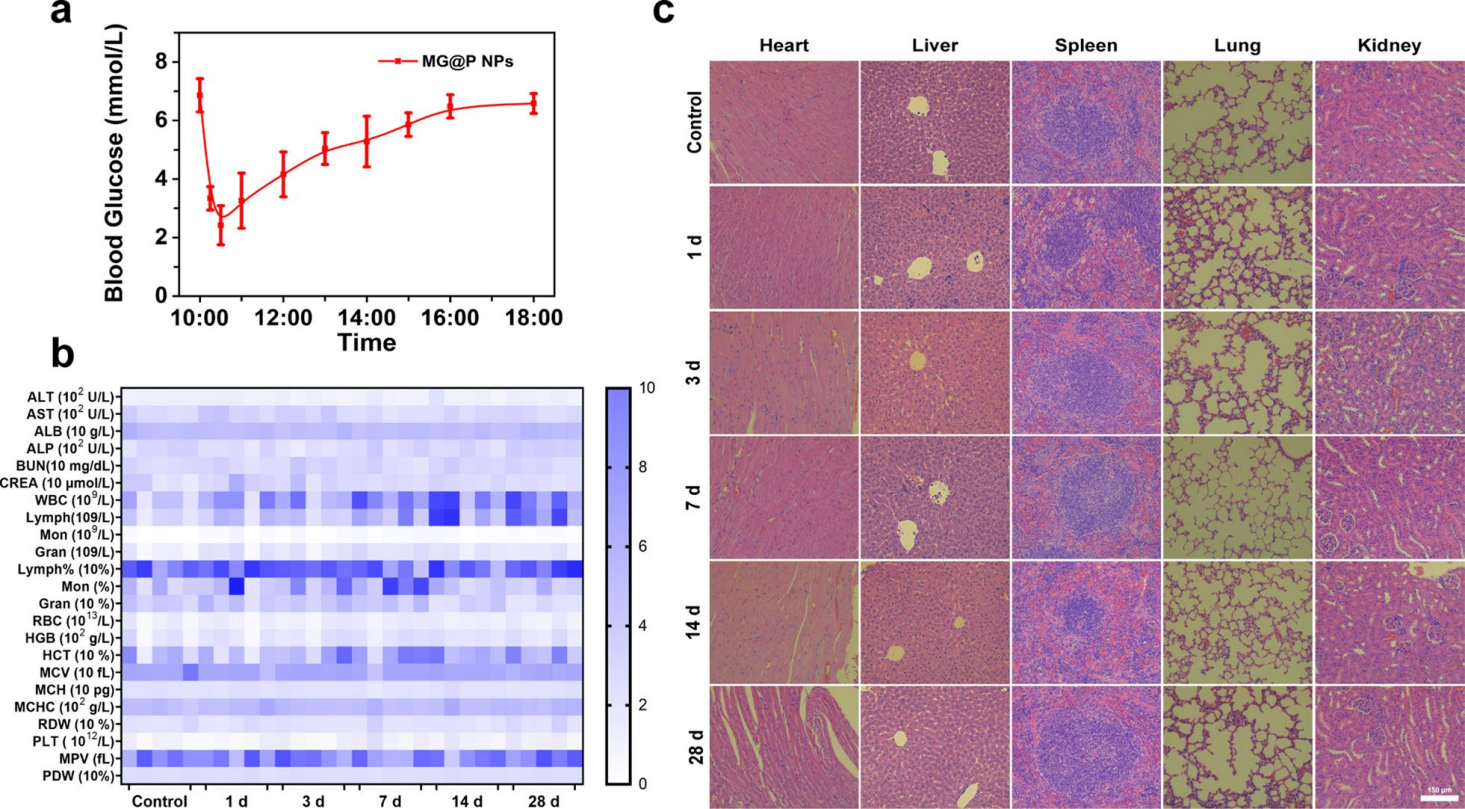
Biosafety assay of MG@P NPs. **a** The blood glucose of mice after intravenously injecting MG@P NPs (0, 0.25, 0.5, 1, 2, 3, 4, 5, 6 and 8 h). **b** Hematological and blood biochemical test of mice after intravenous injection of MG@P NPs (n = 5). **c** Images of H&E staining slices of major organs. The scale bar is 150 μm

The original article has been revised.
