# Supplementary figures and images for: Hypoxia modulation by dual-drug nanoparticles for enhanced synergistic sonodynamic and starvation therapy
Source: J Nanobiotechnology. 2021 Mar 26;19:87. doi: 10.1186/s12951-021-00837-0 (PMC7995598; doi:10.1186/s12951-021-00837-0)

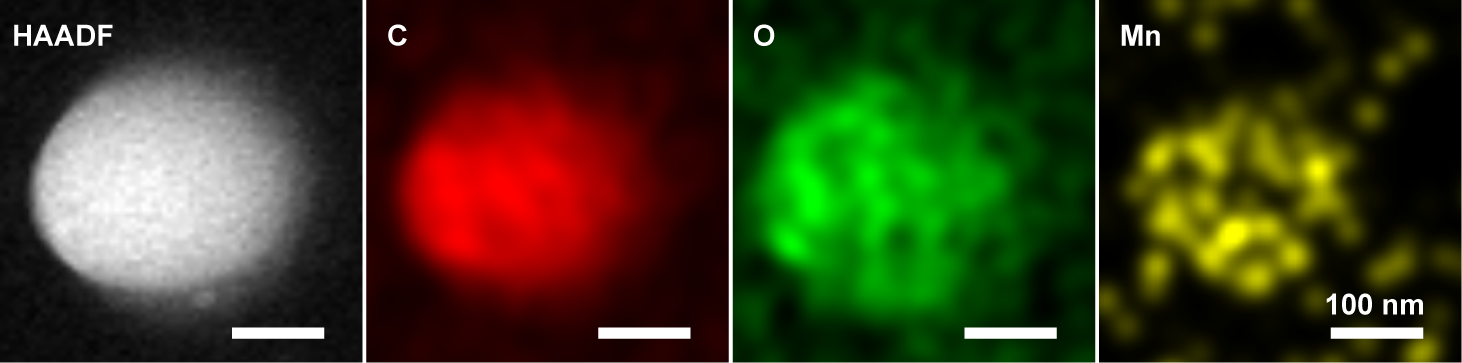

Supplement: Supplementary file 1 — Additional file 1: Figure S1. HAADF-STEM image and area-elemental mappings (C, O and Mn) of MG@P NPs. [file 12951_2021_837_MOESM1_ESM.tif]

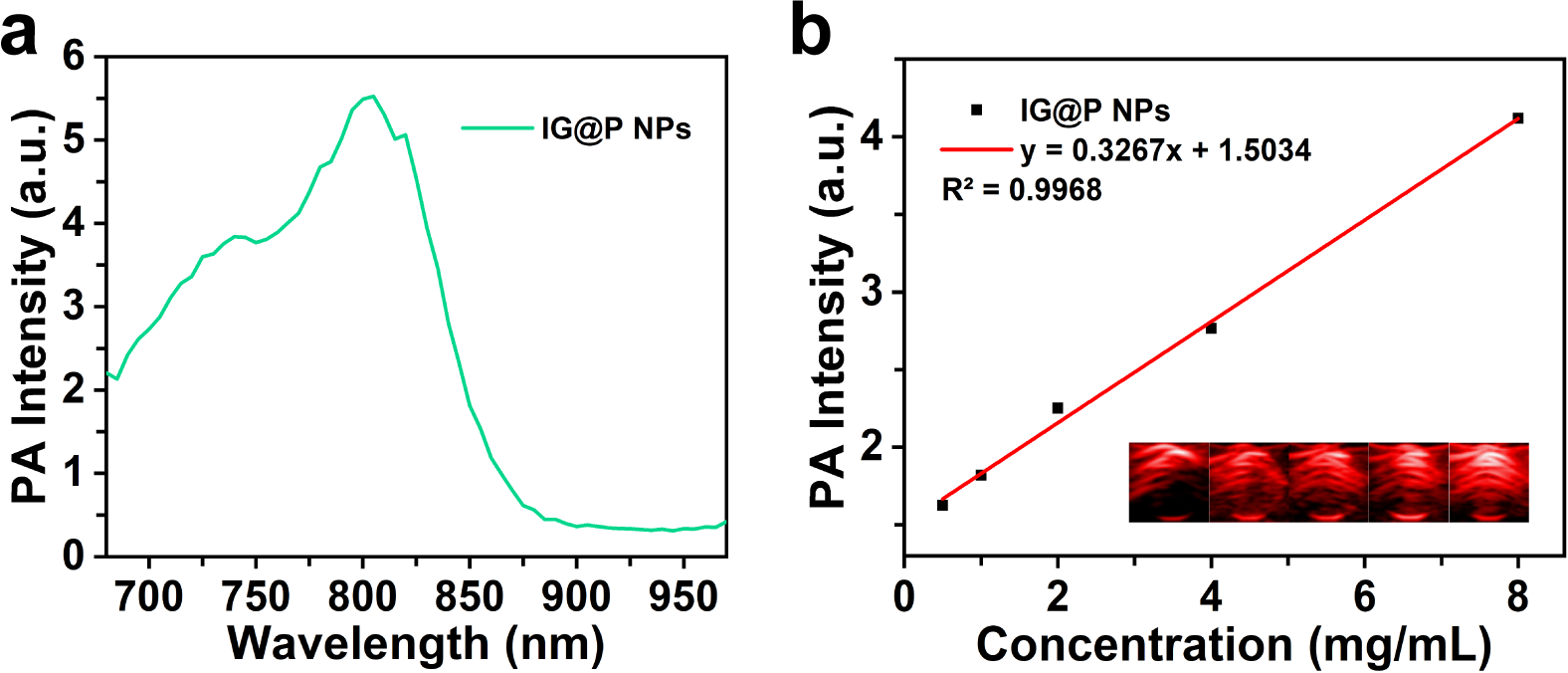

Supplement: Supplementary file 2 — Additional file 2: Figure S2. (a) Quantitative photoacoustic (PA) intensities of I@P NPs in vitro. (b) The linear relationship of PA signal intensity and the concentration of I@P NPs (0.5, 1.0, 2.0, 4.0 and 8.0 mg/mL); the inset was the PA images of I@P NPs aqueous solutions at varied concentrations (λex = 780 nm). [file 12951_2021_837_MOESM2_ESM.tif]
